# Unraveling the Adsorption Mechanism and Anti-Corrosion Functionality of Dextrin and Inulin as Eco-Friendly Biopolymers for the Corrosion of Reinforced Steel in 1.0 M HCl: A Thermodynamic and Kinetic Approach

**DOI:** 10.3390/polym15143144

**Published:** 2023-07-24

**Authors:** Arafat Toghan, Ahmed Fawzy

**Affiliations:** 1 Chemistry Department, College of Science, Imam Mohammad Ibn Saud Islamic University (IMSIU), Riyadh 11623, Saudi Arabia; 2 Chemistry Department, Faculty of Science, South Valley University, Qena 83523, Egypt; 3 Chemistry Department, Faculty of Applied Sciences, Umm Al-Qura University, Makkah 21955, Saudi Arabia; 4 Chemistry Department, Faculty of Science, Assiut University, Assiut 71516, Egypt

**Keywords:** reinforcing steel, eco-friendly corrosion inhibitors, dextrin and inulin biopolymers, adsorption, kinetics

## Abstract

Reinforcing steel (RS) is mainly used in building construction and many industries, but it suffers from corrosion problems, especially in acidic environments. Biopolymers are characterized by their unique chemical composition, as they contain a variety of functional groups that are capable of binding strongly to the metal surface and forming a protective layer on it. Herewith, two biopolymers, viz. dextrin (Dex) and inulin (Inu), were tested as eco-friendly inhibitors for the corrosion of RS in 1.0 M HCl medium at different temperatures. Various experimental tools were utilized in this research. The inhibition efficiencies (% IEs) of the tested polymeric compounds were improved by increasing their doses while reducing with rising temperature. The % IEs of Dex and Inu at a dose of 500 mg/L reached 85% and 93%, respectively. The examined biopolymers displayed cathodic/anodic behavior (mixed type) with a foremost anodic one. The acquired higher % IEs were demonstrated by intense adsorption of Dex and Inu on the RS surface fitting the Langmuir isotherm. The influence of rising temperature in the range of 288–318 K on the corrosion behavior was examined, and the evaluated thermodynamic and kinetic parameters sustained the mechanism of physical adsorption of the polymeric inhibitors. Additionally, the kinetics of corrosion, as well as its inhibition by Dex and Inu, were also investigated. The SEM micrographs of the RS surfaces were accorded with all utilized experimental tools. The results gained from all used tools were discovered to be in good agreement with each other.

## 1. Introduction

Reinforcing steel “rebars” are steel bars that are used with plain cement concrete to obtain reinforced concrete. Rebars have several rewards, such as the capability to withstand the rigors, wearing and tearing through the construction activities, the capability to bend to the wanted specifications, as well as recyclization and reuse for new construction. Rebars are regarded as a significant type of mild steel plain bars. Reinforcing steel in concrete structures, especially those exposed to different environments, is susceptible to corrosion due to many factors, such as pH reduction, carbonation and chloride attack, etc., that result in a reduction in the strength of concrete structures [1,2,3]. Generally, steel corrosion is set to be extremely increased in acidic media, especially hydrochloric acid [4,5,6,7,8,9,10,11,12]. The acidizing procedure in manufacturing cleaning systems of steel removes oxides and/or inorganic layer eliminations [13], and this operation is unavoidable but can be controlled [14]. Therefore, extensive efforts are dedicated to advancing proficient and cost-effectively accommodating ways to reduce steel corrosion [15,16,17,18,19,20,21]. Employment of corrosion inhibitors is regarded as one of such significant ways [22,23,24,25,26]. Corrosion inhibitors are organic compounds comprising electron donor atoms and unsaturated bonds, which allow them to be adsorbed on the metal surface and protect such surfaces from the aggressive media [21,22,23,24,25,26,27,28]. Natural organic compounds are essential kind of ecological inhibitors for metallic corrosion to meet environmental requests [29,30,31,32,33,34]. Additionally, oxygen-rich compounds are desirable inhibitors for corrosion because they are renewable, biodegradable, and environmentally acceptable. Dextrin (Dex) and inulin (Inu) are natural polysaccharides (biopolymers). Dextrin is a mixture of polymer of D-glucose unit connected by α-(1→4) or α-(1→6) glycosidic linkages. It is a low-molecular-weight carbohydrate produced by hydrolysis of starch and glycogen [35]. It is used as water-soluble adhesives, in the mining, foundry, and leather industries, in food processing, coatings, glazes, textile finishing, pharmaceuticals, etc. Inulin is a division of fibers known as fructans that comprise chain-ending glucosyl moieties with a recurring fructosyl moiety connected by β(2,1) linkages [36]. It is produced by various kinds of plants, and it is utilized as a means of storing energy. The two biopolymers, Dex and Inu, have been employed very little as corrosion inhibitors [37,38]. Dextrin was used as a corrosion inhibitor for mild steel in a 15% HCl solution with maximum inhibition efficiency of 84.56% at 0.15 g/L Dex [37]. Inulin was employed for corrosion control of 6061 Al—15%(v) SiC(P) composite in an HCl medium where it showed maximum inhibition efficiency of 88.8% at 1.0 g/L Inu at 303 K [38].

**Figure 1:**
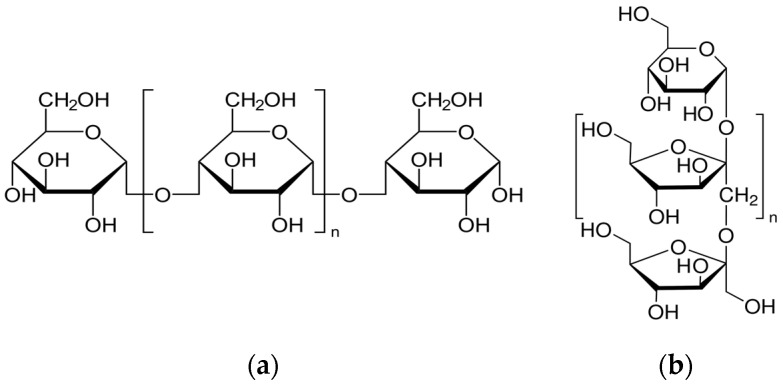
Structures of (**a**) dextrin (Dex) and (**b**) inulin (Inu).

The aim of the present paper is to investigate the effects of two natural biopolymers, viz. dextrin (Dex) and inulin (Inu), whose chemical formulae are illustrated in Figure 1, on the corrosion behavior of reinforcing steel (RS) in 1.0 M HCl solution (corrosive medium) at fixed temperatures. The electrochemical behaviors of RS in the corrosive medium and in the presence of Dex and Inu were studied utilizing both potentiodynamic polarization (PDP) and electrochemical impedance spectroscopy (EIS) tools. Furthermore, the mass-loss (ML) method was employed to evaluate the thermodynamic and kinetic parameters. Finally, the morphologies of steel surfaces were examined by scanning electron microscopy (SEM).

## 2. Experimental Section

### 2.1. Materials

The reinforcing steel (RS) specimens were mild steel plain bars (SABIC company, Riyadh, Saudi Arabia) which were used as the working electrode for the electrochemical experiments (PDP and EIS) and as well as for the mass-loss (ML) experiments with the chemical composition (wt.%): 0.07 C and Si, 0.01 S, 0.02 P, 0.27 Mn, and the rest is iron. The exposed surface area of the RS working electrode for PDP and EIS was 0.95 cm^2^. For ML experiments, each specimen had an exposed surface area of 12.05 cm^2^. Prior to every experiment, silicon carbide sheets with different grades (320 to 1200) were utilized to grind the RS specimens, washed with bidistilled water, degreased with ethanol, and finally dried. The basic corrosive solution (blank) was 1.0 M HCl solution that was prepared by dilution of 37% HCl (Merck, Rahway, NJ, USA) with bidistilled water. Different concentrations (100 to 500 mg/L) of the examined biopolymers inhibitors, dextrin (C_6_H_10_O_5_)*_n_* and inulin (C_6*n*_H_10*n*+2_O_5*n*+1_), were separately added to the blank solution to compare their effects. The corrosion measurements were conducted in non-stirring aerated conditions at fixed temperatures. Each experiment was often repeated about three times to ensure reliability.

### 2.2. Methods

Different methods were utilized in the present work: electrochemical (PDP and EIS), chemical (ML) as well as spectroscopic (SEM).

#### 2.2.1. Electrochemical Methods (PDP and EIS)

Both PDP and EIS were conveyed out in a three-electrode double-jacketed cell with RS as the working electrode, platinum sheet as the counter electrode, and saturated calomel electrode (SCE) as the reference using a PGSTAT30 potentiostat/galvanostat instrument with a temperature-controlled technique. Prior to any experiment, the RS working electrode was prepared, as reported earlier [39,40,41,42]. The RS was dipped in the cell comprising the tested solution (without and with the inhibitor) for a period of time (about 30–40 min) prior to each electrochemical experiment to attain a steady-state circumstance at open circuit potential (OCP). For PDP, the electrode potential was automatically changed at a scan rate of 2.0 mV/s. The EIS measurements were carried out after attaining an OCP value with a 5.0 mV disturbance signal in the frequency range from 100 kHz to 0.1 Hz. 

In PDP, the values of % IEs and the degrees of surface coverage (θ) of the tested compounds were calculated via Equation (1) [42]:

(1) $$\%\;\mathrm{IE}=\theta \;\times \;100=\left[1-\frac{i_{\mathrm{corr}\left(\mathrm{inh}\right)}}{i_{\mathrm{corr}}}\right]\;\times \;100$$

where *i*_corr_ and *i*_corr(inh)_ are corrosion current densities without and with the inhibitor, respectively. 

In EIS, the values of % IEs were calculated using the equation [43]:

(2) $$\%\;\mathrm{IE}=\left[1-\frac{R_{\mathrm{ct}}}{R_{{\mathrm{ct}}_{\left(\mathrm{inh}\right)}}}\right]\;\times \;100$$

where *R*_ct_ and *R*_ct(inh)_ are the charge transfer resistance values without and with the inhibitor, respectively. 

#### 2.2.2. Chemical Method (ML)

The ML experiments were conducted in vessels with temperature control. The RS specimens were bars and were prepared for such experiments, as stated before [31,32]. The prepared RS specimens were weighed before dipping in the tested solutions, then the specimens were removed from the solutions, washed, dried, and weighed. The experiments were performed at various inhibitor doses as well as different temperatures (288–318 K) with an immersion time of 6 h. The corrosion rate (CR) values of RS were evaluated according to Equation (3) [44]:

(3) $$\mathrm{CR}\;(\mathrm{mpy})=\frac{{\mathrm{KM}}_{L}}{\mathrm{Atd}}$$

where *K* is a constant (3.45 × 10^6^), *M*_L_ is the mass-loss (g), *A* is RS specimen area (cm^2^), *t* is time (h), and *d* is the RS density (7.86 g/cm^3^). The values of % IEs and θs of Dex and Inu were computed via Equation (4) [45]:

(4) $$\%\;\mathrm{IE}=\theta \;\times \;100=\left[1-\frac{{\mathrm{CR}}_{\mathrm{inh}}}{\mathrm{CR}}\right]\;\times \;100$$

where CR and CR_inh_ are the corrosion rate values without and with the inhibitor, respectively.

#### 2.2.3. Spectroscopic Method (SEM)

SEM examinations were made using a JEOL SEM model T-200 (Akishima, Tokyo, Japan) with a repeat voltage of 10 kV. SEM micrographs for the surfaces of RS specimens were imaged in order to examine their morphologies prior to and after adding 500 mg/L of the tested inhibitors to verify their effectiveness on the corrosion behavior of RS. Prior to each morphology examination, the RS specimens were cleaned with bidistilled water and dried with N_2_ gas. Then, the dried RS specimens were observed by SEM.

## 3. Results and Discussion

### 3.1. PDP Measurements

**Figure 2:**
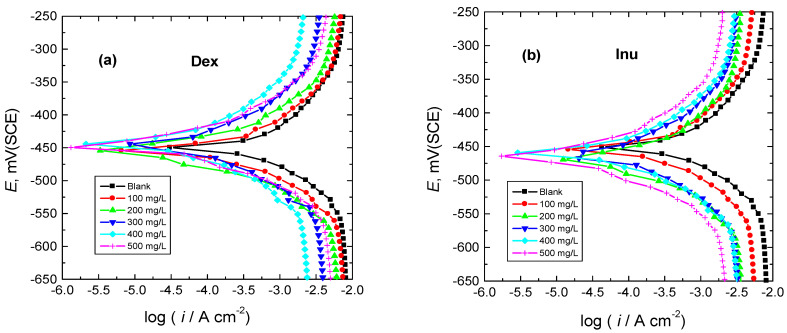
Tafel plots for RS in 1.0 M HCl solution and with: (**a**) Dex and, (**b**) Inu at 298 K.

**Figure 3:**
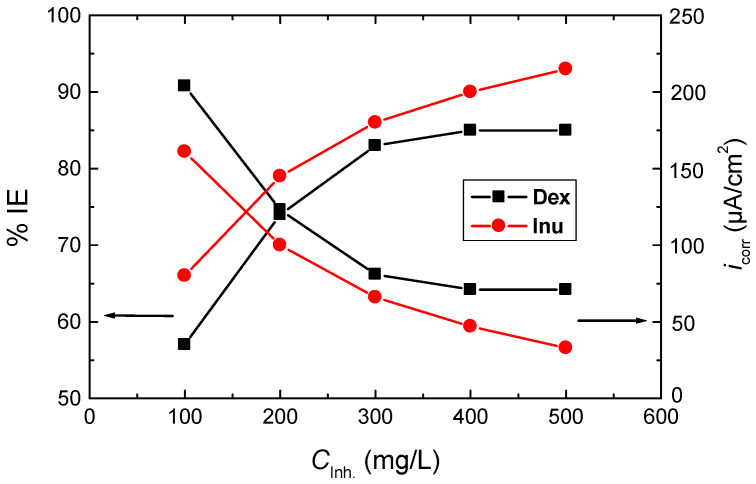
Variation of the values of %IEs and *i*_corr_ with the concentrations of Dex and Inu gained from the PDP results for the corrosion of RS in 1.0 M HCl at 298 K.

The PDP results presented as Tafel plots recorded for RS electrode in 1.0 M HCl solution (blank) and in the presence of different quantities (100–500 mg/L) of Dex and Inu at 298 K are shown in Figure 2. The values of the corresponding corrosion parameters, viz. corrosion potential (*E*_corr_), corrosion current density (*i*_corr_), cathodic and anodic Tafel slopes (*β*_c_, *β*_a_), were derived from such plots as well as the calculated values of polarization resistance (*R*_p_), % IE and θ are inserted in Table 1. Figure 2a,b and the corrosion parameters (Table 1) illuminate that, with the addition of the tested compounds to the blank solution, the PDP (Tafel) curve of the blank solution shifts to smaller current densities, revealing a reduction of RS corrosion rate. The evaluated parameters listed in Table 1 demonstrated that, in comparison with the blank solution, the *E*_corr_ of RS was, in general, somewhat positively shifted upon the addition of Dex and Inu, indicating the mixed-kind nature of the inhibitors alongside a foremost anodic one [46,47]. Values of both *β*_a_ and *β*_c_ in the blank solution were diminished upon adding Dex and Inu, recommending a reduction of the anodic dissolution of RS and hindrance of the cathodic H_2_ evolution [26,27,28]. Additionally, the value of *i*_corr_ of RS in the blank solution was decreased upon raising the concentrations of the tested compounds while the values of % IEs were enhanced, as illustrated in Figure 3.

Inspecting the values of % IEs designated the superiority of Inu over that of Dex at similar concentrations, which can be ascribed to the difference in their molecular structures. In addition, the calculated *R*_p_ value in the blank solution was increased with growing the inhibitors’ concentrations proving the inhibition effects of Dex and Inu. The gained outcomes designated that Dex and Inu are proficient inhibitors for RS corrosion in 1.0 M HCl solution, and this behavior was explained by the adsorption of the inhibitors’ molecules on the RS surface [48].

The explanation of the adsorption mechanism of the examined compounds on the RS surface and their nature were discussed as follows. In acidic solutions, the examined biopolymers (Dex and Inu) are suggested to protonate and become positively charged. Thus, it is essential to compute the potential of zero charges (ZCP) of the examined steel (RS) at the zero point to recognize its surface charge that can be calculated via Equation (5) [49]:

*E*_corr_ − *E*_q_ = 0 (5)

where *E*_corr_ and *E*_q_ are *E*_corr_ and ZCP of Fe, respectively. As mentioned earlier, the *E*_q_ of Fe vs. SCE in the HCl solution was −530 mV [50]. When using Equation (5), if the computed values of Fe-ZCP are larger than zero, the steel surface is suggested to be positively charged [51]. As was listed in Table 1, the *E*_corr_ values recorded at 500 mg/L for both Dex and Inu are −450 and −468 mV, correspondingly. In our present research, the computed values of Fe-ZCP were 80 and 62 mV, correspondingly designating that the surface of the RS steel was positively charged. In addition, in HCl solutions, the surface of the steel is predicted to be covered with Cl^−^ ions, i.e., it became negatively charged. Thus, an electrostatic attraction will be amongst the Cl^-^ ions and the protonated molecules at the metal/medium interface. As a result, Dex and Inu molecules will be attached to the surface of RS via chloride bridges to construct the first adsorbed film. Therefore, the adsorption of the examined compounds on the RS surface will be physical in its nature, constructing an adhesive protective film on its surface, resulting in a decrease in the corrosion rate of RS, as documented in Table 1.

### 3.2. EIS Measurements

**Figure 4:**
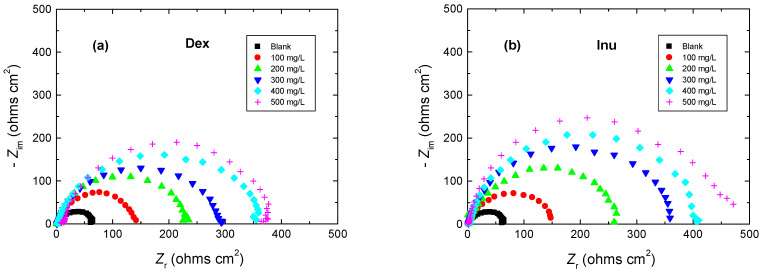
Nyquist plots for the corrosion of RS in 1.0 M HCl solution and with: (**a**) Dex and, (**b**) Inu at 298 K.

In order to obtain more information about the inhibitory effects of the examined biopolymers (Dex and Inu) on the corrosion of RS in 1.0 M HCl solution, EIS measurements were performed. The measurements were recorded after dipping the RS electrode in the tested solutions for about 30 min. or until attaining the OCP. The gained EIS spectra were presented as Nyquist plots shown in Figure 4a,b for Dex and Inu, respectively. The Nyquist plots were recorded for the blank solution and in the presence of various concentrations of the examined compounds at 298 K. Such plots showed single-capacitive semicircles signifying that the molecules of the examined compounds were adsorbed on the RS surface by simple surface coverage and the corrosion of RS is chiefly regulated by charge transfer mechanism [52,53]. The plots shown in Figure 4 indicate that raising the concentrations of Dex and Inu were caused by an increase in the radius of the blank semicircle designating a decrease in the corrosion rate of RS due to the increase in the adsorbed film constructed on the RS surface.

**Figure 5:**
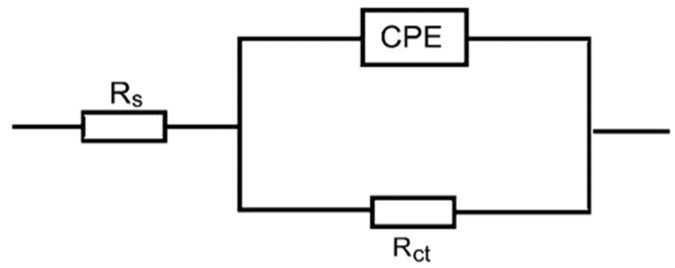
Equivalent circuit employed to suit the obtained EIS spectra for RS in 1.0 M HCl solution and with Dex and Inu.

The gained EIS spectra were analyzed using the equivalent circuit shown in Figure 5, which comprises solution resistance (*R*_s_), charge transfer resistance (*R*_ct_), and constant phase element (CPE). Table 2 includes the fitting results of EIS parameters, which indicates that the addition of the examined compounds to the blank solution significantly increased its *R*_ct_ value. This behavior signifies that the examined compounds were adsorbed at the metal/medium interface leading to a decrease in their electrical capacities due to their displacement of H_2_O molecules and other ions initially adsorbed on the RS surface [54], resulting in an inhibition of the RS corrosion. This is due to the volumes of the inhibitors’ molecules being larger than that of H_2_O molecules, and their dielectric constants are lesser than that of H_2_O molecules leading to increasing in the thickness of the double layer on the RS surface and a reduction in the local dielectric constant. As a result, the value of CPE recorded in the blank solution was discovered to decrease with the increase in the inhibitors’ concentrations, signifying that the inhibitor molecules were effectively adsorbed on the RS surface, which reduces the exposed area of RS and also increases the thickness of the double layer. Consequently, the values of % IEs of Dex and Inu were increased with raising their concentrations, proving that such compounds are proficient inhibitors for the RS corrosion in 1.0 M HCl solution. The results of % IEs gained from both EIS and PDP measurements are set to be chiefly consistent, which illuminated that the values inhibitory effect of Inu is larger than that of Dex at similar concentrations.

### 3.3. ML Measurements

#### 3.3.1. Effect of Inhibitors’ Concentrations

**Figure 6:**
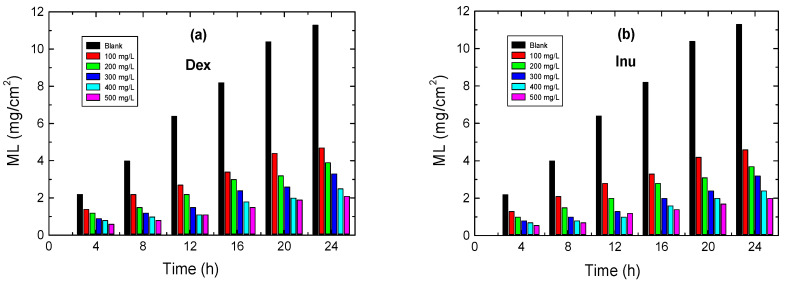
ML vs. immersion time for RS in 1.0 M HCl solution and with: (**a**) Dex and, (**b**) Inu at 298 K.

**Figure 7:**
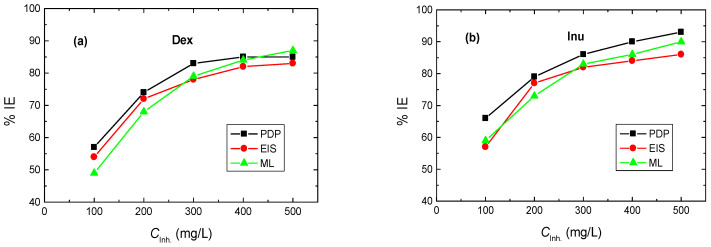
Comparison of % IEs of: (**a**) Dex and, (**b**) Inu, with their concentrations in the corrosion of RS in 1.0 M HCl solution at 298 K using PDP, EIS, and ML techniques.

ML measurements were conveyed to confirm the results gained from both PDP EIS techniques. ML results for RS in 1.0 M HCl solution and with various concentrations of Dex and Inu at 298 K are presented as the mass-loss versus immersion time plots, which are illustrated in Figure 6a,b. From these plots, the values of the corrosion rate (CR in mpy) of RS were calculated using Equation (3) and are inserted in Table 3. In addition, the values of both % IEs and θs of the examined compounds are also computed via Equation (4) and are also listed in Table 4. The data of Table 4 indicates that the CR value of RS in the blank solution decreases, and the values of both % IEs and θs of Dex and Inu increase with rising inhibitors’ concentrations. These outcomes confirm the inhibitory action of such compounds for RS corrosion in 1.0 M HCl solution. In accordance with the results gained from PDP and EIS tools, the order of inhibition efficiencies is Inu > Dex confirming the validity of the outcomes of the employed techniques, as illustrated in Figure 7.

#### 3.3.2. Effect of Temperature

**Figure 8:**
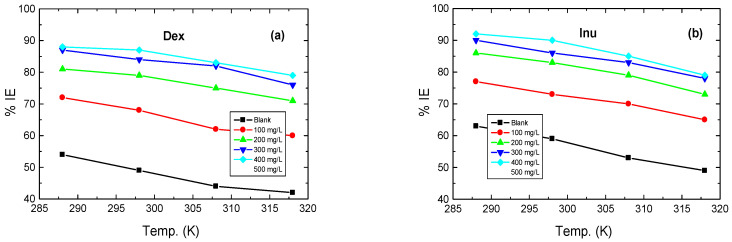
Variation of the values of % IEs of: (a) Dex and, (b) Inu with temperature in the corrosion of RS in 1.0 M HCl.

The effect of rising temperature in the range of 288–318 K on the corrosion behavior of RS in 1.0 M HCl solution and in the presence of the examined compounds was examined using ML measurements in order to evaluate thermodynamic and kinetic parameters and to understand the nature of the inhibitors adsorption on the RS surface. Alike plots illustrated in Figure 6 were obtained but are not shown here, and the related ML parameters are inserted in Table 4. These parameters illuminate that the value of CR of RS increases while those of % IEs of the examined compounds decrease with rising temperature, as illustrated in Figure 8. Decreasing the % IEs values as the temperature rises is related to the acceleration of the H_2_ gas evolution and reduction of the inhibitor adsorption leading to acceleration of the dissolution rate of RS [4]. This behavior proposes that the mechanism of adsorption of the inhibitors’ molecules is physical [43,55,56].

#### 3.3.3. Adsorption Isotherms Examination

**Figure 9:**
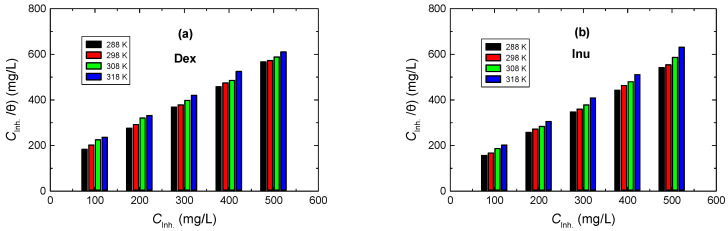
Langmuir adsorption isotherms for: (**a**) Dex and, (**b**) Inu adsorbed on RS surface in 1.0 M HCl solution at different temperatures.

It has been stated [57] that inhibition of metal corrosion by organic molecules is ascribed to the adsorption of such molecules on the metal surface. Adsorption isotherm is a valuable way to suggest the adsorption nature of the examined inhibitors on the metal surface [58]. Therefore, the results (mainly the values of degrees of surface coverage, θ), derived from mass-loss measurements at various temperatures, with respect to inhibitors’ concentrations, were utilized in various adsorption isotherm models (Freundlich, Temkin, Langmuir, Frumkin, etc.) to explain the best-fit isotherm of the investigated inhibitors. Linear plots of *C*_inh_/θ versus inhibitor concentration (*C*_inh_), at different temperatures, with almost unit slopes, were obtained and are presented in Figure 9. These results indicate that the inhibitor adsorption was set to be in good agreement with Langmuir isotherm, represented by Equation (6) [59,60]:

(6) $$\frac{C_{\mathrm{inh}}}{\theta }=\frac{1}{K_{\mathrm{ads}}}+C_{\mathrm{inh}}$$

where *K*_ads_ is the equilibrium constant of the adsorption (listed in Table 4). Indeed, it was reported [10,11,12] that in higher acidic solutions, the Langmuir isotherm model for the adsorption of molecules on the metal surface is suggested to explain the inhibition of metal corrosion. The calculated values of *K*_ads_ were set to decrease with a rising temperature, signifying potent adsorption of the inhibitor molecules on the RS surface at lower temperatures, but at higher ones, the adsorbed molecules tend to desorb from the RS surface.

#### 3.3.4. Thermodynamic Parameters

Thermodynamic parameters regarding the adsorption process were investigated to donate significant information about the mechanism of the corrosion process and its inhibition. The standard free energy (Δ*G*^o^_ads_) was computed at various temperatures using Equation (7) [61]:

Δ*G*^o^_ads_ = −*RT* ln(55.5 *K*_ads_) (7)

Values of Δ*G*^o^_ads_ for Dex and Inu were computed at various temperatures and are inserted in Table 4. The acquired higher values of Δ*G*^o^_ads_ signify that Inu is more effectively adsorbed on the RS surface than the inhibitor Dex. This agrees with the values of % IEs of Dex and Inu gained from all used tools. In addition, the obtained values of Δ*G*^o^_ads_ illuminated that the mechanism of adsorption is physical/chemical adsorption (mixed type) [62,63].

The values of standard heat of adsorption (Δ*H*^o^_ads_) were evaluated via Equation (8) [64]:

(8) $$\ln\;K_{\mathrm{ads}}=\frac{-\Delta H^{o_{\mathrm{ads}}}}{\mathrm{RT}}+\mathrm{Constant}$$

**Figure 10:**
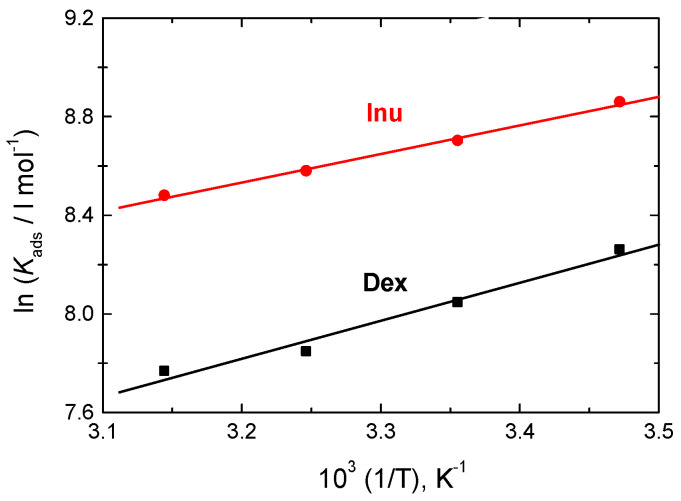
Van’t Hoff plots for Dex and Inu adsorbed on RS surface in 1.0 M HCl solution.

The plots of ln *K*_ads_ vs. 1/T were set to be linear, as shown in Figure 10. From their slopes, the values of ∆*H*^o^_ads_ were gained and are listed in Table 4. The gained negative values of ∆*H*^o^_ads_ suggest that the adsorption process is exothermic with a physical kind (physisorption) [4].

The values of standard entropy of adsorption (∆*S*^o^_ads_) were determined from Equation (9):

∆*G*^o^_ads_ = ∆*H*^o^_ads_ − *T*∆*S*^o^_ads_ (9)

The computed values of ∆*S*^o^_ads_ (Table 4) showed an increase in the randomness (disorder) at the metal/medium interface through the adsorption of inhibitors’ molecules on the RS surface. Such an increase in disorder may be due to the desorption of more H_2_O molecules from the RS surface and their replacement by inhibitors’ molecules [65].

#### 3.3.5. Kinetic Parameters

**Figure 11:**
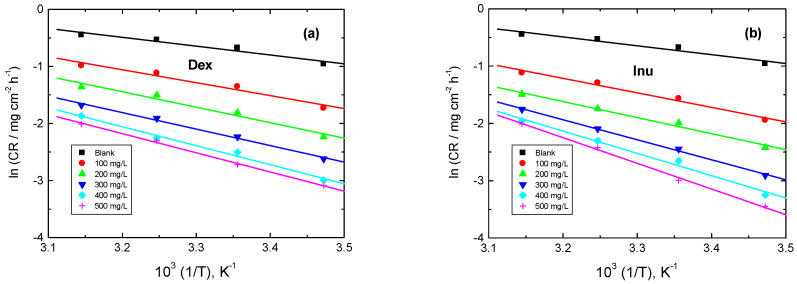
Arrhenius plots for RS corrosion in 1.0 M HCl solution and with: (**a**) Dex and, (**b**) Inu.

The relation between the CR and temperature is expressed by the Arrhenius equation, Equation (10) [66]:

(10) $$\ln\;\mathrm{CR}=\ln\;A-\frac{{E_{a^{}}}^{*}}{\mathrm{RT}}$$

where *E*_a_^*^ is the activation energy. The plots of ln CR vs. 1/*T* are illustrated in Figure 11. From these plots, the values of *E*_a_^*^ were computed and are inserted in Table 5. The gained *E*_a_^*^ values in the presence of Dex and Inu were greater than that obtained in the blank. These findings signify the adsorption of Dex and Inu on the RS surface, constructing a barrier to separate such surface from the corrosive solution [67]. The values of *E*_a_^*^ were smaller than 80 kJ/mol that required for chemical adsorption, indicating that the kind of adsorption was physical [68]. These outcomes are in agreement with those based on Δ*G^o^*_ads_ and Δ*H^o^*_ads_ values, signifying the validity of the gained results.

The enthalpy of activation (ΔH^*^) and entropy of activation (ΔS^*^) are evaluated via Equation (11) [69]:

(11) $$\ln\left(\frac{\mathrm{CR}}{T}\right)=\left(\ln\frac{R}{\mathrm{Nh}}+\frac{\Delta S^{*}}{R}\right)-\frac{\Delta H^{*}}{R}\frac{1}{T}$$

**Figure 12:**
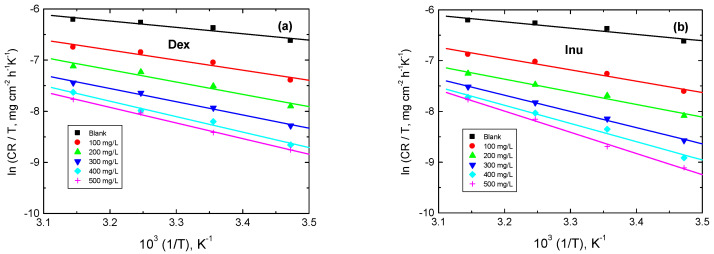
Transition state plots for RS corrosion in 1.0 M HCl solution and with: (**a**) Dex and, (**b**) Inu.

The plots of ln(CR/*T*) vs. 1/*T* were set to straight (Figure 12). The evaluated values of ΔH^*^ and ΔS^*^ are listed in Table 5. The gained positive values of ΔH^*^ propose that the corrosion process was endothermic, where the negative values of ΔS^*^ in the blank solution and with the examined biopolymers illuminate a high reduction in the randomness due to the formation of activated complexes [70].

#### 3.3.6. Kinetics of Corrosion and Its Inhibition

**Figure 13:**
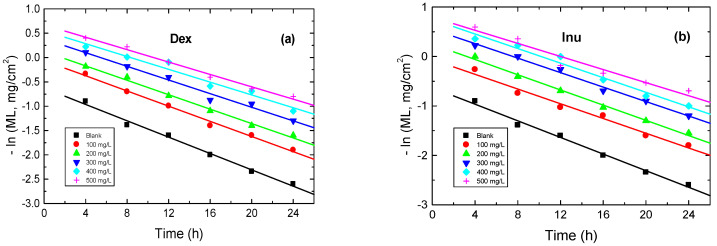
First-order plots for RS corrosion in 1.0 M HCl solution and with: (**a**) Dex and, (**b**) Inu at 298 K.

The kinetics of RS corrosion in 1.0 M HCl solution and with various concentrations of Dex and Inu were studied. In this context, the plots of –ln(ML) vs. time were linear, as illustrated in Figure 13, signifying that the kinetics of RS corrosion in 1.0 M HCl solution and its inhibition by Dex and Inu were negatively first-order processes. The slopes of such plots refer to the first-order rate constant values, *k*_1_, that are inserted in Table 6. Additionally, the values of half-life times, *t*_1/2_, were calculated (Table 6) via the following equation [71]:

(12) $$t_{1/2}=\frac{0.693}{k_{1}}$$

In addition, the order (*n*) of corrosion inhibition of RS by Dex and Inu was evaluated using Equation (13) [72]:

log CR = log *k* + n log *C*_inh._ (13)

where *k* is the specific rate constant.

**Figure 14:**
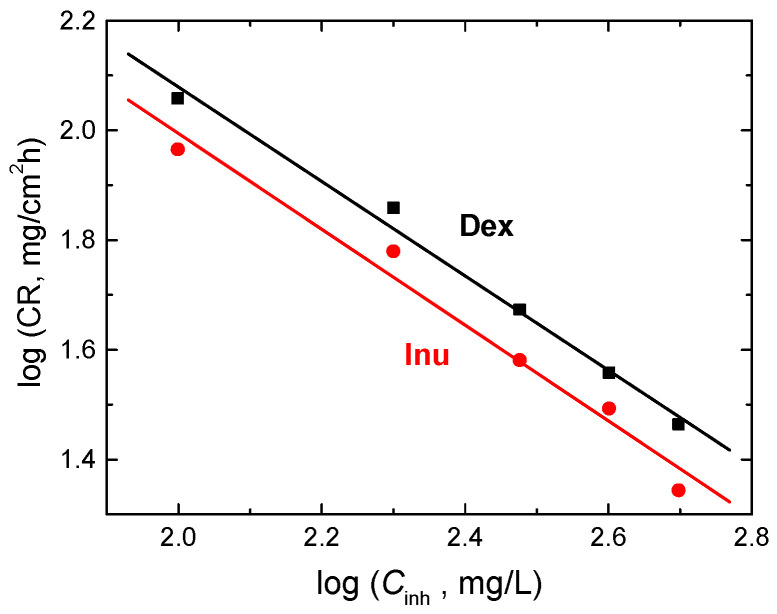
Log CR vs. log *C*_inh_ for inhibition of RS corrosion in 1.0 M HCl solution by Dex and Inu at 298 K.

The plots of log CR vs. log *C*_inh_ for Dex and Inu at 298 K were linear, as shown in Figure 14. Values of *n* were calculated and were found to be −0.86 and −0.89 for Dex and Inu, respectively. The acquired values of *n* suggest that the corrosion inhibition process is a negative fractional-first-order reaction with respect to *C*_inh_. The negative *n* values and the opposite proportionality of the CRs with *C*_inh_ (Figure 14) indicate good % IEs of the examined compounds [73].

### 3.4. SEM Examinations

**Figure 15:**
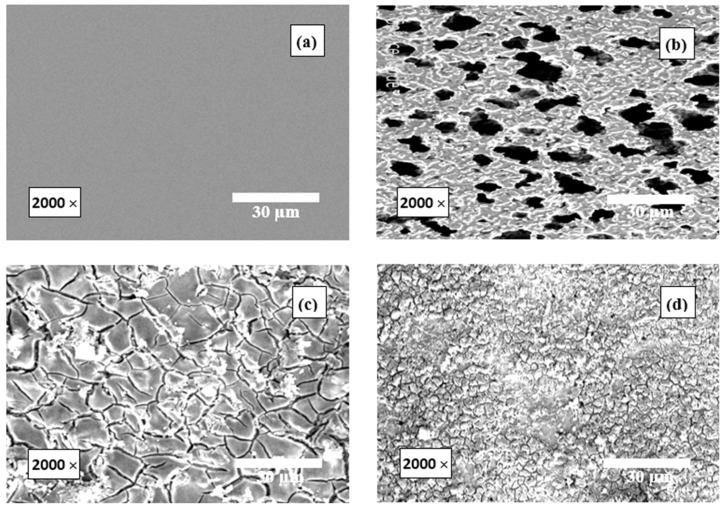
SEM images for the surfaces of RS in 1.0 M HCl solution: (**a**) before immersion, (**b**) after 12 h immersion, and (**c**,**d**) in the presence of 500 mg/L of Dex and Inu, respectively.

The morphologies of the surfaces of RS specimens in 1.0 M HCl solution and in the presence of 500 mg/L of the examined compounds (Dex and Inu) are shown in Figure 15a–d. Figure 15a,b shows the polished RS surfaces before and after 12 h immersion in the blank solution, respectively. Figure 15b shows the appearance of a large number of corrosion pits on the RS surface. Figure 15c,d, in the presence of 500 mg/L of Dex and Inu, respectively, show a noteworthy change in the RS surface where the corrosion pits shown in the RS surface disappeared, and the surface was chiefly covered with the inhibitor molecules on the whole surface. This could be ascribed to the effective adsorption of the molecules of the examined compounds on the RS surface, constructing an adhesive layer that protects the surface from the corrosive solution, and displaying outstanding inhibition properties. Thus, the SEM micrographs of the RS surfaces were set to accord with the various utilized experimental tools.

## 4. Conclusions

1.   Two biopolymers, dextrin (Dex) and inulin (Inu), were tested as inhibitors for the corrosion of reinforcing steel (RS) in 1.0 M HCl using various experimental tools.
2.   The inhibition efficiencies (% IEs) of the tested biopolymers were improved by augmenting their doses while reducing with rising temperature.
3.   The % IEs of Dex and Inu at a dose of 500 mg/L reached 85% and 93%, respectively.
4.   The tested biopolymers displayed mixed type with a foremost anodic one.
5.   The acquired high % IEs were demonstrated by intense adsorption of Dex and Inu on the RS surface fitting the Langmuir isotherm.
6.   The influence of rising temperature in the range of 288–318 K was examined.
7.   Thermodynamic and kinetic parameters sustained the mechanism of physical adsorption of the inhibitors.
8.   The kinetics of corrosion and its inhibition by Dex and Inu were also investigated.
9.   The SEM results were set to accord with the various utilized experimental tools.
10. The results gained from all used tools were discovered to be in good agreement with each other.

## Figures and Tables

**Table 1:** PDP parameters for RS in 1.0 M HCl solution and with Dex and Inu at 298 K.

1.0 M HCl +	Inh. Conc. (mg/L)	*−E*_corr_ (mV(SCE))	*β*_a_ (mV/dec.)	*−β*_c_ (mV/dec.)	*i*_corr_ (µA/cm^2^)	*R*_p_ (ohm cm^2^)	% IE	θ
**-**	**0**	**451**	**109**	**88**	**474**	**45**	**-**	**-**
**Dex**	**100**	456	102	76	204	93	57	0.57
	**200**	459	103	78	123	157	74	0.74
	**300**	447	99	73	81	226	83	0.83
	**400**	445	96	72	71	252	85	0.85
	**500**	450	95	81	71	268	85	0.85
**Inu**	**100**	450	105	85	161	127	66	0.66
	**200**	464	97	83	100	194	79	0.79
	**300**	461	104	77	66	291	86	0.86
	**400**	459	94	75	47	386	90	0.90
	**500**	468	97	79	33	567	93	0.93

**Table 2:** EIS parameters for RS in 1.0 M HCl solution and with Dex and Inu at 298 K.

1.0 M HCl +	Inh. Conc. (mg/L)	*R*_s_ (ohm cm^2^)	*R*_ct_ (ohm cm^2^)	10^−2^ CPE (µF/cm^2^)	% IE	θ
**-**	**0**	**2.73**	**66**	**29.07**	**-**	**-**
**Dex**	**100**	1.69	144	13.15	54	0.54
	**200**	2.31	236	10.34	72	0.72
	**300**	3.40	300	9.31	78	0.78
	**400**	4.97	367	8.21	82	0.82
	**500**	7.23	388	7.37	83	0.83
**Inu**	**100**	3.05	154	12.76	57	0.57
	**200**	2.16	287	9.66	77	0.77
	**300**	0.82	367	8.14	82	0.82
	**400**	4.07	413	7.51	84	0.84
	**500**	3.42	471	7.06	86	0.86

**Table 3:** ML data for RS in 1.0 M HCl solution and with Dex and Inu at different temperatures.

1.0 M HCl +	Inh. Conc. (mg/L)	Temperature (°K)											
		288			298			308			318		
		CR (mpy)	% IE	θ	CR (mpy)	% IE	θ	CR (mpy)	% IE	θ	CR (mpy)	% IE	θ
**-**	**0**	**169**	**-**	**-**	**224**	**-**	**-**	**258**	**-**	**-**	**282**	**-**	**-**
**Dex**	**100**	78	54	0.54	114	49	0.49	144	44	0.44	164	42	0.42
	**200**	47	72	0.72	72	68	0.68	98	62	0.62	113	60	0.60
	**300**	32	81	0.81	47	79	0.79	65	75	0.75	82	71	0.71
	**400**	22	87	0.87	36	84	0.84	46	82	0.82	68	76	0.76
	**500**	20	88	0.88	29	87	0.87	44	83	0.83	59	79	0.79
**Inu**	**100**	63	63	0.63	92	59	0.59	121	53	0.53	144	49	0.49
	**200**	39	77	0.77	60	73	0.73	77	70	0.70	99	65	0.65
	**300**	24	86	0.86	38	83	0.83	54	79	0.79	76	73	0.73
	**400**	17	90	0.90	31	86	0.86	44	83	0.83	62	78	0.78
	**500**	14	92	0.92	22	90	0.90	39	85	0.85	59	79	0.79

**Table 4:** Values of thermodynamic parameters and *K*_ads_ for RS corrosion in 1.0 M HCl solution and with Dex and Inu at different temperatures.

1.0 M HCl +	Temp. (°K)	10^−3^ *K*_ads_ L mol^−1^	∆*G*^o^_ads_ kJ mol^−1^	∆*H*^o^_ads_ kJ mol^−1^	∆*S*^o^_ads (298)_ J mol^−1^ K^−1^
**Dex**	**288**	3.86	−29.39	−12.82	57.53
	**298**	3.12	−29.82		57.05
	**308**	2.56	−30.32		56.82
	**318**	2.36	−31.17		57.70
**Inu**	**288**	7.03	−30.82	−9.64	73.54
	**298**	6.01	−31.45		73.19
	**308**	5.31	−32.19		73.21
	**318**	4.81	−33.06		73.65

**Table 5:** Activation parameters for RS corrosion in 1.0 M HCl solution and with Dex and Inu.

1.0 M HCl +	Inh. Conc. (mg/L)	*E*_a_^*^ kJ mol^−1^	∆H^*^ kJ mol^−1^	∆S^*^ J mol^−1^ K^−1^
**-**	**0**	**12.88**	**10.35**	**−81.10**
**Dex**	**100**	18.87	16.39	−95.67
	**200**	22.53	20.01	−95.59
	**300**	24.02	21.55	−94.01
	**400**	27.69	25.12	−84.03
	**500**	27.93	25.37	−91.10
**Inu**	**100**	21.03	18.69	−98.17
	**200**	23.28	20.80	−94.84
	**300**	29.09	26.54	−79.02
	**400**	32.42	29.78	−69.86
	**500**	37.07	34.69	−55.29

**Table 6:** Values of *k*_1_ and *t*_1/2_ for RS corrosion in 1.0 M HCl solution and with Dex and Inu at 298 K.

Inh. Conc. (mg/L)	Dex		Inu	
	10^3^ *k*_1_, h^−1^	*t*_1/2_, h	10^3^ *k*_1_, h^−1^	*t*_1/2_, h
**0**	**84**	**8.25**	**84**	**8.25**
**100**	77	9.01	74	9.36
**200**	74	9.36	76	9.12
**300**	70	9.90	73	9.49
**400**	66	10.51	72	9.63
**500**	63	11.02	66	10.50

## Data Availability

The data that support the findings of this study are available on request from the corresponding author.
